# GBsim: A Robust GCN-BERT Approach for Cross-Architecture Binary Code Similarity Analysis

**DOI:** 10.3390/e27040392

**Published:** 2025-04-07

**Authors:** Jiang Du, Qiang Wei, Yisen Wang, Xingyu Bai

**Affiliations:** School of Cyber Science and Engineering, Information Engineering University, Zhengzhou 450001, China

**Keywords:** graph neural network robustness, binary code similarity analysis, cross-architecture embedding, hybrid deep learning

## Abstract

Recent advances in graph neural networks have transformed structural pattern learning in domains ranging from social network analysis to biomolecular modeling. Nevertheless, practical deployments in mission-critical scenarios such as binary code similarity detection face two fundamental obstacles: first, the inherent noise in graph construction processes exemplified by incomplete control flow edges during binary function recovery; second, the substantial distribution discrepancies caused by cross-architecture instruction set variations. Conventional GNN architectures demonstrate severe performance degradation under such low signal-to-noise ratio conditions and cross-domain operational environments, particularly in security-sensitive vulnerability identification tasks where feature instability or domain shifts could trigger critical false judgments. To address these challenges, we propose GBsim, a novel approach that combines graph neural networks with natural language processing. GBsim employs a cross-architecture language model to transform binary functions into semantic graphs, leverages a multilayer GCN for structural feature extraction, and employs a Transformer layer to integrate semantic information, generates robust cross-architecture embeddings that maintain high performance despite significant distribution shifts. Extensive experiments on a large-scale cross-architecture dataset show that GBsim achieves an MRR of 0.901 and a Recall@1 of 0.831, outperforming state-of-the-art methods. In real-world vulnerability detection tasks, GBsim achieves an average recall rate of 81.3% on a 1-day vulnerability dataset, demonstrating its practical effectiveness in identifying security threats and outperforming existing methods by 2.1%. This performance advantage stems from GBsim’s ability to maximize information preservation across architectural boundaries, enhancing model robustness in the presence of noise and distribution shifts.

## 1. Introduction

Binary Code Similarity Analysis (BCSA) is a significant and challenging technology. It can effectively identify and match binary functions, enabling the detection of code reuse and potential security threats. It has already played a crucial role in application scenarios that include vulnerability discovery [1], malicious code detection and classification [2], software plagiarism detection [3], and patch analysis [4]. With the extensive use of open source software and the common occurrence of code reuse, the importance of BCSA has become increasingly critical, providing essential support for maintaining software security. However, given the increasingly complex software ecosystem, the development of robust and accurate BCSA methods has become a pressing need.

In recent years, the rapid advancements in deep learning technology have introduced innovative solutions for BCSA. Numerous studies have focused on the use of deep learning models for the extraction of features and comparison of binary functions, successfully identifying similar binary functions between different compilers, optimization levels, and Instruction Set Architectures (ISA), even under certain obfuscation techniques [5,6,7,8,9,10]. These efforts underscore the potential for deep learning in this domain. However, despite notable progress, current deep learning-based BCSA methods still face significant challenges in practical applications, particularly in cross-architecture scenarios, where performance bottlenecks are especially evident.

Firstly, the representation of binary functions across different architectures poses a critical robustness challenge for graph neural networks (GNNs) in BCSA. Different instruction set architectures represent distinct data distributions with varying instruction sets, register usage patterns, and calling conventions. This architectural diversity creates a fundamental distribution shift problem, where GNNs often struggle to maintain consistent performance. Existing methods typically rely on features specific to a single architecture or expert-defined heuristics, severely limiting their robustness and generalization capabilities when analyzing binary code across different architectures. Consequently, developing graph representations with enhanced cross-architecture robustness remains a formidable challenge in this domain.

Secondly, in the context of GNN applications for BCSA, a significant challenge lies in understanding the relationship between model complexity and resilience to architectural variations. While more complex GNN architectures theoretically offer greater representational capacity, they may also be more susceptible to overfitting architecture-specific patterns rather than capturing the underlying structural similarities that persist across different instruction sets. Our experiments demonstrate this non-linear relationship between model complexity and cross-architecture performance, highlighting the importance of balancing model capacity with generalization capabilities when developing robust graph-based approaches for binary similarity analysis.

Lastly, in the task of BCSA, a critical challenge lies in the trade-off between efficiency and accuracy during the comparison phase, particularly in the choice of comparison strategies. Researchers often encounter a scenario where they are presented with a library of binary functions that have already been embedded and a newly acquired binary function that has yet to be embedded. The task at hand is to generate the embedding representation of the new function using the same method and then perform similarity comparisons within the function library. In this one-to-many search scenario, the performance and computational cost of the comparison algorithm play a crucial role, even more so than the vectorization method of binary functions. The design of the comparison algorithm directly impacts the efficiency and accuracy of the search process, especially in large-scale function libraries, where its effectiveness determines the feasibility of practical applications. It is obvious that existing methods struggle to balance accuracy and efficiency well [11]. When building models, comparison-based BCSA methods [12,13,14] use a pair of binary functions as the key input and carefully analyze their similarity. Although it is true that this can ensure relatively high accuracy, the resulting high computational cost cannot be ignored. In contrast, embedding-based BCSA methods [5,8,10,15,16,17,18] work on a different principle. They take only a single binary code as input and use special encoding strategies to turn the advanced features of the binary code into an embedding space. Subsequently, vector distance metrics such as the cosine distance are employed to approximately evaluate the similarity of function pairs within this space. Since each input function only needs to be encoded once and is combined with the fast neighbor search algorithm, these methods have great scalability and can quickly process large amounts of data. However, compared with comparison-based methods, embedding-based methods, which focus only on extracting features from a single function, exhibit noticeably lower accuracy.

With the aim of overcoming the hurdles outlined earlier, this paper puts forward a cross-architecture binary function embedding and comparison approach underpinned by a hybrid layer network.

To address the challenge of cross-architecture binary representation, this paper proposes a construction method of a cross-architecture assembly language model based on word vectors. The control flow graph (CFG) of a binary function is regarded as a sequence of sentences. Then, natural language processing (NLP) techniques are employed to generate word vectors for each instruction. The sentence vectors of the basic blocks are generated by average pooling, and these basic block vectors serve as node features. Moreover, the relationships among basic blocks are depicted by the edges of the graph, thereby constructing a cross-architecture binary function representation. Furthermore, a hybrid layer network composed of GCN [19] and BERT [20] is used to extract structural and semantic features from binary functions, generating robust cross-architecture embeddings. This innovative approach addresses the issue of inconsistent binary code representations caused by architectural differences, providing a unified and robust way to handle cross-architecture scenarios. The motivation for choosing the GCN+BERT combination lies in enhancing graph representation robustness against architectural variations and structural noise while demonstrating the power of foundational models. Rather than pursuing complex state-of-the-art architectures, we deliberately selected the pioneering GCN model from the GNN domain and the pioneering BERT model from the NLP domain as representative approaches to handle structural and semantic information, respectively. This choice was guided by the hypothesis that even these early influential models, when effectively combined to leverage both structural and semantic information, could achieve robust cross-architecture performance. Indeed, our experimental results confirm that this approach reaches state-of-the-art (SOTA) levels even with these foundational models, demonstrating that model combination strategy can be more important than model complexity for achieving robustness. This not only validates our approach but also indicates significant room for improvement when more advanced GNN and transformer models are incorporated within our framework, offering promising directions for future research on robust graph representation learning.

With regard to the challenge of understanding the relationship between model complexity and performance, this paper conducts comprehensive investigations. By varying the complexity of the language model and the hybrid layer network (composed of GCN and BERT), we systematically study the impact of model complexity on performance. The experimental results demonstrate that increasing model complexity does not always lead to significant performance improvements and may even degrade performance in some cases. Through adjusting the number of GCN and BERT layers as hyperparameters, we validate the non-linear relationship between model complexity and performance, providing insights into selecting the optimal model complexity for practical applications. This approach ensures a balance between model complexity and performance while acknowledging the inherent trade-off with efficiency.

In response to the challenge of balancing accuracy and efficiency during the comparison phase, a two-stage search algorithm is devised. In the initial stage, cosine distances are utilized to preliminarily select candidates for obviously dissimilar functions during the function search process, eliminating options with significant differences. Subsequently, a more accurate comparison-based method is employed to complete the final comparison. This approach combines the efficiency of the embedding-based method in the first stage and the high accuracy of the comparison-based method in the final stage, effectively reconciling the trade-off between accuracy and efficiency in the BCSA domain.

In summary, our contributions are as follows.

- We propose a cross-architecture assembly language model construction method and design a GNN-based binary function embedding approach that demonstrates enhanced robustness to architectural variations, addressing the fundamental challenge of creating reliable graph representations across different instruction set architectures.
- We systematically investigate the relationship between GNN complexity and cross-architecture performance through comparative experiments on language models of varying complexities and hyperparameter configurations, revealing key insights into developing more robust graph models for binary similarity analysis.
- We develop a two-stage search algorithm that enhances both the accuracy and efficiency of graph-based binary code similarity analysis, effectively balancing the trade-off between performance and computational cost in practical security applications.
- We make GBSim available to the research community at https://github.com/kidding1412/GBsim, facilitating further research on robust GNNs for binary code analysis.

## 2. Background

BCSA presents significant challenges for GNNs, especially in terms of robustness. Unlike standard graph applications, binary code graphs exhibit extreme structural variations and feature inconsistencies across different compilation settings, forming an ideal testbed for evaluating and improving GNN robustness. The robustness challenges in BCSA stem primarily from two sources: cross-architecture compilation and varying optimization levels, which introduce substantial distributional shifts in both graph structure and node features.

In the work of binary code similarity analysis, cross-architecture and cross-optimization are crucial elements that cannot be overlooked. When compiling the source code, it first goes through a compiler that parses and checks it. Then, depending on the architecture of the target instruction set and the chosen optimization level, the compiler transforms the source code into binary functions, a process where cross-architecture and cross-optimization can greatly impact the outcome.

### 2.1. Cross-Architecture Perspective

**Figure 1:**
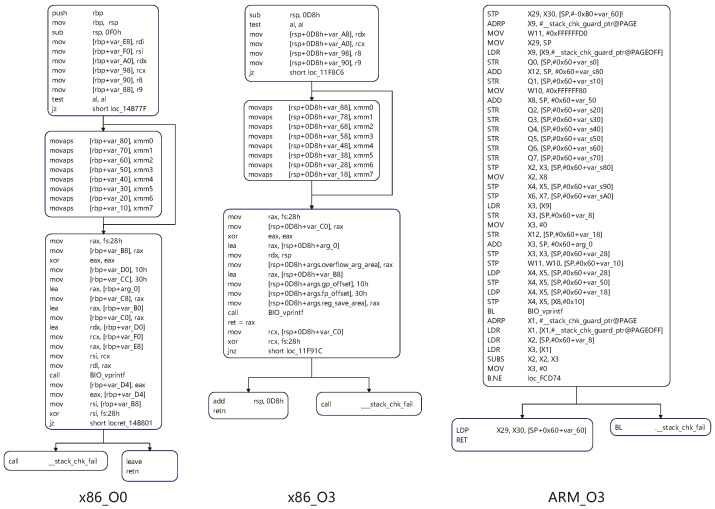
Impact of cross-architecture and cross-optimization on the binary code of BIO_printf function.

Different instruction set architectures, such as x86 and ARM, have a profound impact on the final generated binary code due to significant differences in instruction sets, register usage, and calling conventions. Taking the BIO_printf function in the OpenSSL library as an example, as depicted in Figure 1, the CFG of the binary function compiled with O0 optimization under the x86 architecture shows significant structural differences compared to that of the binary function compiled with O3 optimization under the ARM architecture. The characteristics of the x86 instruction set determine that its binary code exhibits specific node connections and process flows in the CFG. In contrast, due to the uniqueness of its own instruction set, the corresponding CFG of the ARM architecture is completely different in structure, with obvious distinctions in aspects such as node composition and branch distribution, which fully reflects the structural changes brought about by cross-architecture to binary code.

These structural transformations across architectures create a fundamental robustness challenge for graph neural networks. When a GNN is trained on graphs from one architecture and tested on another, it encounters a significant distributional shift that conventional GNN models struggle to handle. This represents a practical manifestation of the out-of-distribution generalization problem widely discussed in robust machine learning research.

### 2.2. Cross-Optimization Perspective

Even within the same architecture (such as x86), different optimization levels (such as O0 and O3) can cause significant differences in binary code. It can be clearly observed from the Figure 1 that the binary code of the BIO_printf function compiled with O0 optimization under the x86 architecture has a relatively intuitive and simple instruction sequence, manifested as a more regular process and fewer optimization traces in the CFG. However, when compiled with the O3 optimization level, the instructions of the binary code undergo significant changes. Some redundant instructions are eliminated, and the execution order and combination of instructions are optimized. This not only leads to changes at the instruction level but also makes the structure of the binary function present a more compact and complex form in the CFG, such as the merging of certain nodes and the simplification of branches. These differences caused by cross-architecture and cross-optimization pose numerous challenges to binary code similarity analysis work, and also highlight the necessity and importance of an in-depth exploration of these factors in this research field.

From a GNN robustness perspective, these optimization-induced transformations introduce structural noise and feature instability that test the limits of graph neural networks’ generalization capabilities. A truly robust GNN must maintain consistent performance despite these significant variations in graph topology and node attributes—a challenge that mirrors the broader problems of graph perturbation robustness and structural noise resistance in graph machine learning research.

## 3. Related Work

BCSA is a technique used to identify similarities between binary code fragments, such as functions. The core principle involves extracting features from binary code (e.g., semantic and structural features) and leveraging these features for similarity measurement, enabling applications in vulnerability detection, malware classification, and code plagiarism detection. With the advancement of deep learning techniques, BCSA has made significant progress in feature extraction and similarity computation, allowing for the automatic learning of complex code features and improving the accuracy of analysis.

### 3.1. Classification Based on Features

Existing BCSA methods can be broadly categorized into three types based on the deep learning models they employ: semantic feature-based methods, structural feature-based methods, and hybrid methods that combine semantic and structural features.

1. Semantic feature-based methods. These methods analyze instruction sequences using NLP techniques to extract the functional semantics of binary code. For example, binary instructions are treated as words in a sentence, and models such as Word2Vec [21], RNN, LSTM, or BERT [20] are utilized to capture the contextual semantics of instructions [6,9,15]. However, these methods often overlook structural information, leading to suboptimal performance in cross-architecture function representation. The significant differences in instruction sets across architectures make it challenging to capture cross-architecture similarities solely based on semantic features.
2. Strructural feature-based methods. These methods focus on the structural characteristics of binary code, such as CFG and abstract syntax trees (ASTs) [22], and use GNNs [8,14,23,24] to capture the execution logic and flow of the program. Nevertheless, these methods often rely on expert knowledge during feature extraction, such as selecting call information or the in-degree/out-degree of basic blocks in CFGs as node features. This reliance can introduce bias and limit the adaptability to diverse code characteristics.
3. Hybrid methods. These methods aim to integrate the strengths of both semantic and structural features by combining the semantics of the instruction with the information about the structure of the code, allowing for a more complete representation of the binary code [10,11,17,25]. While these hybrid approaches demonstrate promising performance, their reliance on joint structural-semantic features introduces new robustness challenges under distribution shifts (e.g., unseen compiler environments). Recent advancements in graph Out-Of-Distribution (OOD) detection, such as GOODAT [26], provide methodological insights to improve generalization across architectural boundaries by identifying invariant subgraph patterns. However, hybrid methods typically require complex models to separately extract semantic and structural information, resulting in high computational costs. Moreover, existing research rarely discusses the trade-offs between model complexity and performance, which restricts their scalability in practical applications.

### 3.2. Classification Based on Comparison Approaches

Based on the comparison approach, the BCSA methods can be divided into two categories: embedding-based methods and comparison-based methods.

1. Embedding-based methods. These methods [8,10,15,17,27] map binary functions into a low-dimensional embedding space using deep learning models and compute similarity using distance metrics (e.g., cosine distance). The primary advantage of embedding-based methods lies in their ability to precompute embeddings, making them suitable for large-scale applications. However, their accuracy is often limited because similarity computation relies solely on distance metrics between embedding vectors, which may fail to capture complex inter-function relationships.
2. Comparison-based methods. These methods [12,13,14] directly analyze raw data or features of two binary functions and compute similarity using deep learning models such as CNNs, LSTMs, or attention mechanisms. Comparison-based methods generally achieve higher accuracy, as they can directly model complex relationships between functions. However, they incur higher computational costs, particularly when processing large-scale datasets, making them less efficient for real-time or large-scale inference.

## 4. GBsim Approach

**Figure 2:**
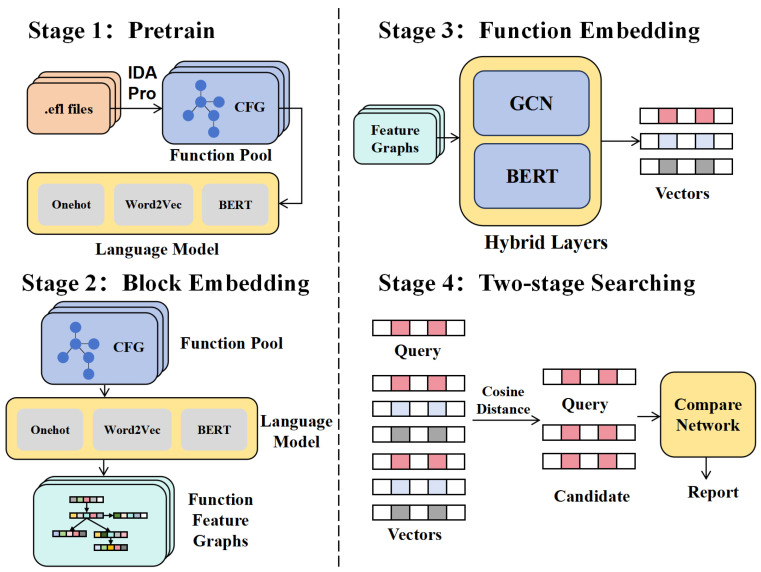
GBsim workflow. This figure depicts the four-stage methodology for binary code analysis, including Pretrain, Block Embedding, Function Embedding, and Two-Stage Searching.

This paper proposes a novel approach to analyzing binary code similarity, which consists of four distinct stages. As illustrated in Figure 2, these stages form a comprehensive pipeline that aims to address the challenges in this domain. Stage 1 involves the pretraining process of the language model, establishing its fundamental capabilities. In Stage 2, the pretrained language model specifically transforms CFGs of binary functions into function feature graphs through pattern recognition and semantic analysis. Stage 3 employs a hybrid neural network architecture to convert these function feature graphs into vectorized embedding representations. Finally, Stage 4 performs similarity measurement through vector space search operations. The following sections will dive into the details of each stage, elucidating their functionalities and contributions to the overall methodology.

### 4.1. Pretraining

Within the research domain of cross-architecture code similarity analysis, achieving an effective representation of binary functions across different architectures poses a central challenge. Essentially, it lies in accurately discerning and capturing the commonalities and differences inherent in the function characteristics under different ISAs. To address this predicament, we meticulously devised a language model with the ability to capture syntactic and semantic similarities among diverse ISAs, enhancing the robustness of our approach to distribution shifts between architectures.

Specifically, with regard to the CFG of binary functions, we consider the content of basic blocks as independent sentence units, and thus the CFG composed of numerous basic blocks was perceived as a paragraph made up of multiple sentences. Based on this unique perspective, we initiated the construction of a corpus, aiming to provide solid data support for the subsequent training of the language model.

Prior to formal model construction, the data standardization process to circumvent the Out-Of-Vocabulary (OOV) issue is of paramount importance. For the operands in assembly instructions, including those of special types and those exhibiting multiple combination forms, such as immediate values, addresses, variable names, function names, etc., we specifically designed and implemented a special token replacement strategy to fulfill the objective of unified and standardized processing.

This standardization process is crucial from an information theory perspective, as it effectively reduces the entropy in the representation space by removing irrelevant variations, thus creating a more robust foundation for cross-architecture analysis.

To evaluate the impact of model complexity on BCSA performance, we concurrently constructed language models using the onehot, Word2vec, and BERT methods. To ensure compatibility with onehot encoding, we uniformly set the word vector dimension to 128, as the vocabulary size after standardization was determined to be 117. When employing the Word2vec model, we directly adopted its default parameter settings and precisely adjusted the window size to 5. Meanwhile, when opting for the BERT model, we accurately selected the BERT-based parameter combination.

### 4.2. Block Embedding

Upon completion of the pretraining process, we obtained an assembly language model capable of being simultaneously compatible with different ISAs. Subsequently, we performed basic block embedding through the assembly language model to derive robust function feature graphs that maintain consistent representations across architectural boundaries.

**Figure 3:**
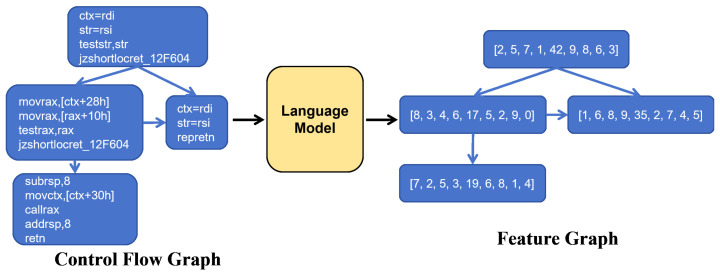
The process of deriving function feature graphs from assembly language model.

To compare the impact of language models with different complexities on the performance of BCSA in subsequent experiments, we respectively utilized three types of language models to obtain three corresponding function feature graphs. Firstly, we regarded each basic block within the CFG of the binary function as a sentence and replaced each word in the sentence with the word vector obtained from the corresponding language model. Secondly, we performed average pooling on the sentences to aggregate and obtain a vector representing the basic block. Finally, within the CFG, we replaced the corresponding basic block with this vector while keeping the graph structure intact. In this way, we successfully obtained the feature graphs of the functions. The specific process is illustrated in Figure 3.

The multilayer hybrid architecture is specifically designed to enhance the model’s robustness against noisy graph structures commonly encountered in binary code analysis. By combining GCN’s ability to capture structural patterns with BERT’s semantic understanding, this design creates complementary representations that remain stable even when individual aspects of the code (either structure or semantics) are affected by noise or architectural variations.

### 4.3. Function Embedding

**Figure 4:**
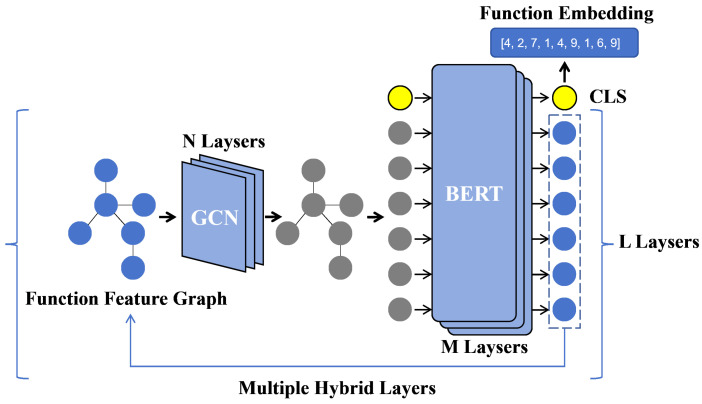
Multilayer hybrid network. The multilayer hybrid network combines GCNs with the BERT model and accomplishes the extraction of high-quality embeddings for binary functions by means of multilayer stacking.

After obtaining the feature graph of a binary function, we need to vectorize it to generate the embedding representation of the function. As illustrated in Figure 4, we propose a multilayer hybrid network architecture that integrates the GCN and BERT models, combining structural and semantic information to generate embeddings for binary functions. Our design is motivated by two principles: (1) the emerging consensus in binary code analysis prioritizes the joint learning of structural (GNNs) and semantic (NLP) features, and (2) foundational architectures like GCN and BERT provide robust baselines to validate core ideas while resisting over-engineering. Specifically, a single hybrid layer consists of n GCN layers and m BERT layers, and the entire hybrid network comprises l such layers. Here, n, m, and l are hyperparameters of the GBsim model, with each parameter ranging from 1 to 3. To evaluate the relationship between model complexity and performance, we performed extensive testing on different combinations of n, m, and l to explore the optimal configuration.

The GCN module updates node features based on the CFG. Let the control flow graph be represented as a graph G=(V,E), where *V* denotes the set of nodes and *E* denotes the set of edges. Each node represents a basic block. The GCN layer updates node representations by aggregating information from neighboring nodes, as described by the following equation:

(1) $$\begin{array}{c} H^{\left(l+1\right)}=\sigma \left(D^{-\frac{1}{2}}AD^{-\frac{1}{2}}H^{\left(l\right)}W^{\left(l\right)}\right) \end{array}$$

Here, $H^{\left(l\right)}$ represents the node feature matrix at the *l*-th layer, $H^{\left(0\right)}$ denotes the initial node features, *A* is the adjacency matrix of the graph, *D* is the degree matrix of *A*, $W^{\left(l\right)}$ is the learnable weight matrix in the *l*-th layer, and σ is the activation function, such as ReLU. This equation captures the structural information of the control flow graph by aggregating information from neighboring nodes into the central node through graph convolution.

This equation captures the structural information of the control flow graph by aggregating information from neighboring nodes into the central node through graph convolution. From a robustness perspective, this aggregation process helps mitigate the impact of isolated structural anomalies that might appear in binary functions due to compilation differences or partial recovery of control flow edges.

After passing through the GCN module, the sequence of node features is fed into the BERT layers. To better adapt the BERT model to our framework, several modifications were made:

- Removal of the embedding layer: The original embedding layer in BERT was removed because all embedding operations were already handled by the prior function feature graph generation. The resulting feature sequence can directly serve as input to the BERT network layers.
- Elimination of the Next Sentence Prediction (NSP) task: The NSP task, designed for natural language contexts to help the model understand inter-sentence relationships, was removed. In our scenario, each function is treated as an independent “sentence” input to BERT, and thus the concept of a “next sentence” does not logically apply. Moreover, previous studies [28] have shown that removing the NSP task can improve the performance of the model and simplify the computation in many cases. Therefore, the NSP task was excluded from this model.

The modified BERT layer processes sequence information in a way that is invariant to specific instruction set architectures, focusing instead on the underlying semantics of the operations. This architectural choice significantly contributes to the model’s robustness when handling cross-architecture binary code.

The BERT layer processes the serialized basic block features and generates embeddings at the function level. Let the node feature sequence after GCN processing be denoted as $\left\{h_{1},h_{2},\cdots ,h_{n}\right\}$, which is entered into the BERT model’s word embedding layers. The operation of the BERT layer can be described by the following equation:

(2) $$\begin{array}{c} E_{i}=\mathrm{BERT}\left(h_{1},h_{2},\ldots ,h_{n}\right), \end{array}$$

where $E_{i}$ represents the semantic embedding of the *i*-th basic block after the BERT encoding layer. If BERT is the last layer, its output CLS vector $E_{\mathrm{cls}}$ is used as the final embedding of the function. If BERT’s output is not the last layer, its result will be used to update the node features in the function feature graph and then continuously input to GCN for cyclic processing.

### 4.4. Two-Stage Searching

In this study, the two-stage search algorithm plays a crucial role in balancing search robustness and efficiency when comparing the similarity between binary functions. In the first stage, the cosine distance is calculated between the query function and all functions in the search pool, and the k nearest top results are selected as the candidate set. This initial filtering provides a robust mechanism against outliers and noise in the search space.

In the second stage, a two-layer fully connected network is employed for a fine comparison. A single-layer fully connected network is not chosen because when mapping the 256-dimensional input vector (formed by concatenating two 128-dimensional vectors) to 1 dimension, it essentially computes the inner product with a 256 × 1 weight sequence, which is prone to information loss. Although fully connected three-layer and above networks can handle complex relationships, they would significantly increase the complexity of the model and the time cost, making them unsuitable for large-scale tasks.

The input of this two-layer fully connected network is a 1 × 256 concatenated vector. The first layer reduces the 256 dimensions to 128 dimensions to reduce redundancy and retain key information. After being activated by ReLU, the second layer further reduces the 128 dimensions to 1 dimension to output a similarity value. Finally, the Sigmoid function is used to map the result in the range of 0 to 1, making the similarity score intuitive and comparable.

### 4.5. Training

Within the GBsim framework, three crucial training procedures are mainly involved. The first of these is the pretraining stage of the language model, whose corresponding details have been elaborately expounded in the “pretraining” chapter.

Secondly, attention is focused on the training phase of the multilayer hybrid network. During this process, we adopt TripletMarginLoss as the loss function, with its key parameter, the margin, precisely set to 1.0, and apply it to the triplet structure composed of the original function, the positive function, and the negative function. This loss function can effectively drive the similarity between the original function and the positive function in the embedding space to approach the maximum, while making every effort to minimize the similarity with the negative function.

Third, emphasis is placed on the training of the two-stage comparison network. Given that this network is specifically applied to search task scenarios, objective conditions make it difficult for us to construct an ideal environment where the ratio of positive and negative examples is strictly balanced at 1:1. In view of this, we employ the cross-entropy loss function commonly used in classification tasks. Essentially, this task aims to perform a binary classification on candidate functions one by one.

## 5. Experimental Setup

### 5.1. Baseline Comparisons

For this particular study, we have meticulously handpicked one exemplary baseline approach from each of the three distinct BCSA technology pathways elucidated in Section 2. These pathways, namely, methods based on semantic features, methods based on functional structural features, and methods that combine semantic and structural features, each offer unique perspectives and techniques in the realm of binary code similarity analysis.

Our focus was not only on the novelty and effectiveness of these baselines, but also on their reproducibility and reliability. To this end, we deliberately chose baselines that have emerged from top academic conferences, ensuring that they have been rigorously vetted by the research community. Moreover, the availability of comprehensive official code implementations was a key determinant, as it allowed us to minimize human-induced errors and discrepancies during the replication process, thereby maintaining the highest standards of scientific integrity.

SAFE [15]: This particular method falls under the umbrella of those based on semantic features. At its core, SAFE harnesses the power of an RNN (Recurrent Neural Network) architecture augmented with attention mechanisms. By taking assembly instructions as input, it is capable of generating a highly representative and meaningful embedding of the analyzed function. In our implementation, we adhered scrupulously to its official PyTorch codebase, ensuring that every line of code was faithfully replicated. Throughout our evaluation phase, the default parameter settings provided by the original authors were maintained, guaranteeing an apples-to-apples comparison and a reliable assessment of its performance.

Asteria-Pro [22]: As a representative of methods predicated on functional structural features, Asteria-Pro exhibits a remarkable ability to exploit structural information to its fullest potential. During the critical stages of 1-to-N searching, rapid prefiltering, and the encoding of the function’s AST (Abstract Syntax Tree), Asteria-Pro demonstrates a deep understanding and utilization of the inherent structural nuances within the code. Our implementation of Asteria-Pro was based on its official code, with an unwavering commitment to the default parameter settings. This ensured that we captured the essence of the method as intended by its creators, allowing for a fair and accurate comparison.

jTrans [17]: Positioned at the intersection of semantic and structural features, jTrans represents a novel amalgamation of ideas. It ingeniously adapts the concept of unique positional vectors, a hallmark innovation of BERT-based NLP techniques, to decipher and interpret jump information within functions. This adaptation empowers sequence-oriented natural language models to process and make sense of the structural jumps that are an integral part of code functions. In our implementation, we relied on the official jTrans source code, leveraging the pretrained models available in the official repository.

### 5.2. Hardware and Software Environment

The experiments were carried out on an Ubuntu 22.04 system using Python 3.10 and PyTorch 2.1.0, with CUDA 12.1 for GPU acceleration. The hardware was as follows:

One Nvidia L40 GPU with 48GB memory to accelerate matrix operations in GCN and BERT layers, significantly reducing training time.

An AMD EPYC 9K84 96-Core Processor with 16 virtual CPUs for data preprocessing and non-GPU computations.

A total of 150 GB RAM, ensuring smooth data loading and processing of large datasets during training.

### 5.3. Dataset

We validated our model using the BINKIT [29] dataset, a comprehensive dataset designed for tasks related to binary codes. It contains 243,128 binary files and 36,256,322 functions, generated from 51 software packages in 1352 combinations of compiler configurations, optimization settings, and target architectures (x86, ARM, MIPS). The dataset spans eight processor architectures and multiple versions, considering the impact of diverse compiler options on binary code.

For evaluation, we sampled 50,000 positive and 50,000 negative function pairs (100,000 pairs total) from all architectures within BINKIT. Data were split into training, validation, and test sets in a ratio of 8:1:1. This division ensured broad feature coverage in the training set while retaining sufficient validation and test samples for robust model tuning and performance evaluation.

In the function search task, we also constructed a function pool composed of 10,000 functions from the BINKIT dataset, which was used to evaluate the performance of the model in binary function search tasks. To evaluate the model’s performance in cross-optimization binary function search tasks, we also constructed a dedicated search dataset specifically designed for cross-optimization scenarios. In particular, this is a purely testing dataset, and no training has been conducted on it for any of the work.

The BINKIT dataset represents an ideal testbed for evaluating model robustness under real-world distribution shifts. The diversity of compiler configurations, optimization settings, and target architectures creates natural challenges that mirror practical scenarios where binary code analysis must remain robust despite significant variations in how source code is compiled.

### 5.4. Evaluation Metrics

We adopted MRR and Recall@1 as the primary evaluation metrics. These are highly effective in ranking quality in retrieval tasks. To assess the impact of hyperparameter complexity on performance, we also employed accuracy for quick and comparative analysis.

In binary software similarity analysis, real-world applications typically involve one-to-many retrievals. Although traditional metrics such as ROC-AUC and accuracy are valuable for evaluating binary classification and serve as foundational measures for one-to-many retrieval, they have limitations in directly reflecting retrieval quality in such scenarios.

MRR evaluates the reciprocal rank of the positive example in the set of candidates, precisely measuring the quality of the ranking of positive examples.

Recall@1 focuses on whether the positive example is correctly ranked first in the candidate set, a critical metric for determining the model’s ability to swiftly and accurately retrieve the target.

These metrics align closely with real-world applications of binary software similarity analysis, providing a direct and reliable assessment of the model’s performance.

## 6. Evaluation

To validate the effectiveness and robustness of the GBsim model in addressing the numerous challenges previously highlighted in the BCSA domain, particularly those related to graph structure noise and cross-architecture distribution shifts, we designed a series of experiments to answer the following research questions:

- RQ1: How does model complexity impact performance?
- RQ2: How does GBsim compare to SOTA solutions in terms of performance?
- RQ3: How does GBsim perform in real-world vulnerability search scenarios?
- RQ4: How does GBsim’s inference time cost compare to baseline models?
- RQ5: How does the two-stage search algorithm affect the trade-off between accuracy and efficiency?

These questions aim to comprehensively evaluate GBsim’s capabilities across multiple critical dimensions, including performance trade-offs, practical applicability, and efficiency in real-world use cases.

### 6.1. Hyperparameter Sensitivity and Robustness Analysis (RQ1)

To evaluate the impact of model complexity on performance and robustness against distribution shifts, we conducted experiments in models with varying numbers of GCN layers, BERT layers, and hybrid layers, and different word embedding pretraining strategies. The GCN, BERT, and hybrid layers were varied from 1 to 3, while word embeddings were tested with three schemes: onehot, Word2Vec, and BERT. Accuracy was used as an evaluation metric for model performance across different hyperparameters, as it provides a simple and effective comparison in scenarios with numerous straightforward evaluations. A total of 81 hyperparameter configurations were tested.

**Figure 5:**
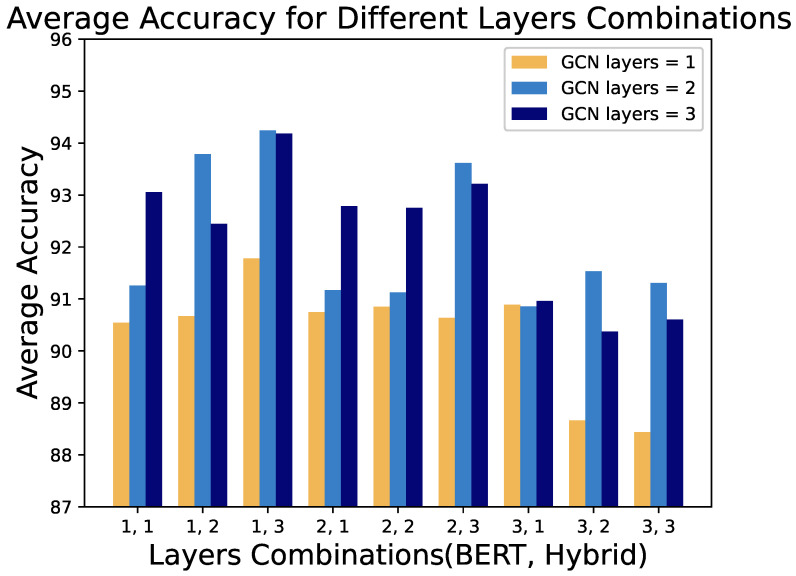
Different layers combinations by GCN layers. The labels on the x-axis (e.g., ‘1,1’) represent combinations of BERT layers and Hybrid layers, where the first number is the number of BERT layers and the second number is the number of Hybrid layers.

Figure 5 illustrates the performance of the models in various combinations of depths of the deep learning layer. Each bar represents the average accuracy of the three embedding schemes for a given combination. From the figure, we observe that a GCN depth of 1 consistently yields the worst performance across configurations. Theoretically, a single-layer GCN only captures the information of one-hop neighbors, which inadequately represents the structural features of binary function CFGs. As model complexity increases, GCN’s contribution to performance is generally positive. However, in all configurations with three hybrid layers, models with two GCN layers outperform those with three layers, indicating diminishing returns with additional GCN layers in highly complex networks. Models with a single-layer GCN in high-complexity configurations consistently perform the worst.

This observation has significant implications for model robustness in cross-architecture scenarios, as it suggests that finding the optimal GCN depth is crucial for extracting structural features that remain stable across different architectures while avoiding overfitting to architecture-specific graph patterns.

**Figure 6:**
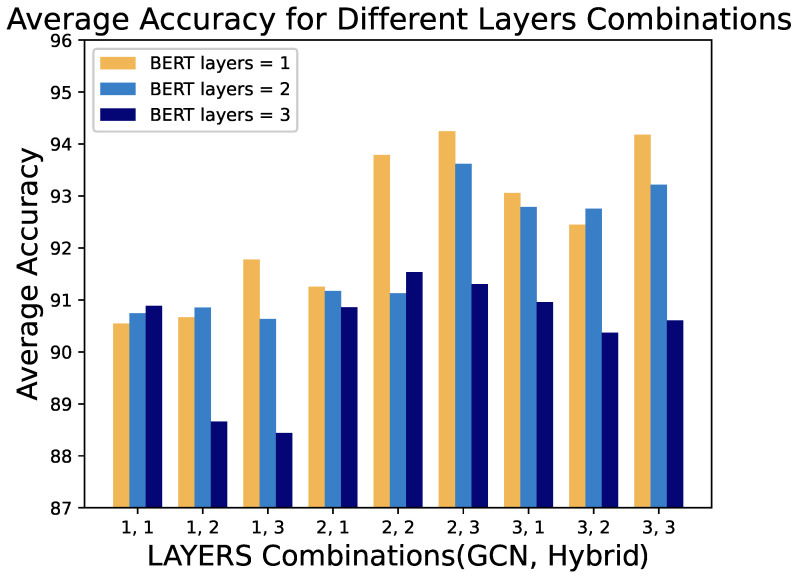
Different layers combinations by BERT layers. The labels on the x-axis (e.g., ‘1,1’) represent combinations of GCN layers and Hybrid layers, where the first number is the number of GCN layers and the second number is the number of Hybrid layers.

Figure 6 examines the impact of BERT layers on performance in different combinations of GCN and hybrid layers. It is evident that as model complexity increases, single-layer BERT models exhibit improved performance. However, when the GCN depth is 1, models with three BERT layers perform poorly. This suggests that stacking additional BERT layers does not compensate for the lack of sufficient structural information in the features. In contrast, with adequate structural information, a single BERT layer achieves excellent performance, reflecting efficient utilization of available features.

These results demonstrate the complex interplay between structural and semantic robustness in our model. While BERT layers can enhance semantic understanding, their effectiveness is contingent upon having sufficiently robust structural representations from the GCN layers, highlighting the importance of balanced feature extraction for cross-architecture robustness.

**Figure 7:**
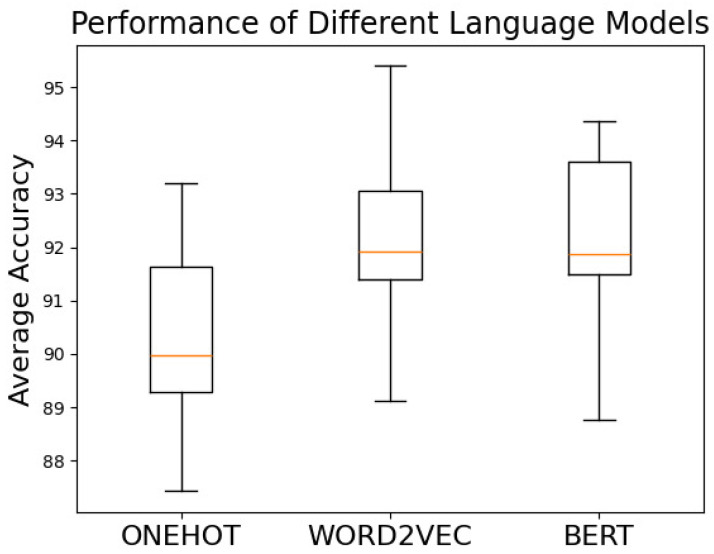
Performance of different language models.

Figure 7 presents boxplots of performance across all network depth configurations for different word embedding schemes, showing maximum, minimum, quartiles, and medians. The onehot embedding consistently performs the worst, as it essentially relies on statistical features derived from average pooling of basic block representations, which is weaker in extracting semantic features compared to Word2Vec and BERT. The median performance of BERT is almost identical to that of Word2Vec, with the interquartile range of BERT outperforming that of Word2Vec. However, the best results are achieved with Word2Vec, which also has a higher minimum performance than the other two models. Although BERT is considered the next-generation model over Word2Vec in NLP, addressing issues like polysemy and dynamic context, these advantages are less relevant in assembly language. Assembly code, being a precise programming language, inherently avoids polysemy. Additionally, standardized preprocessing eliminates extensive vocabulary size, making the advantages of BERT in natural language processing less impactful for assembly code embeddings.

These findings highlight an important observation regarding robustness in binary code analysis: simpler language models like Word2Vec may sometimes offer greater stability across distribution shifts, as they focus on capturing core semantics while avoiding overfitting to architecture-specific patterns that do not generalize well across different instruction sets.

In the subsequent evaluation, we will conduct experiments using the best-performing GBsim configuration, which consists of a Word2Vec embedding model, a two-layer GCN, a one-layer BERT, and a three-layer hybrid architecture.

### 6.2. Comparison with Baseline Models: Assessing Cross-Architecture Robustness (RQ2)

**Figure 8:**
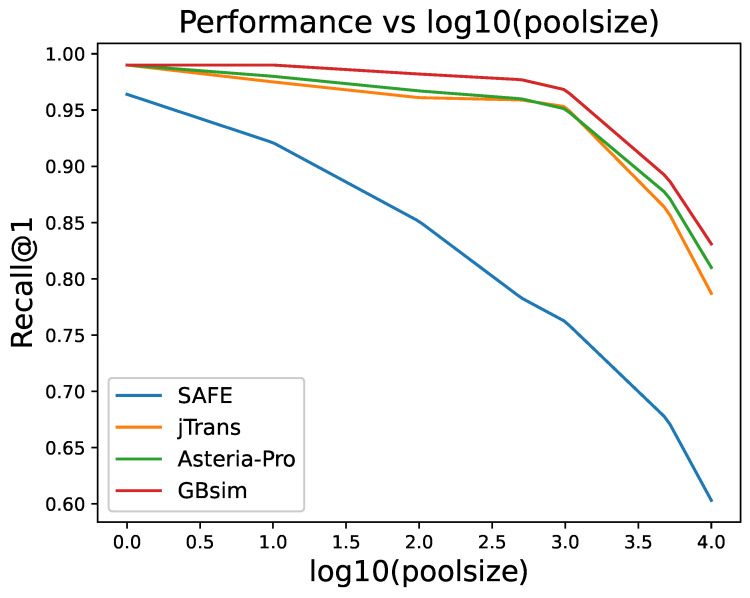
Performance of GBsim and baseline models.

In this experiment, we evaluated the performance of the GBsim model and the baseline models in binary function search tasks within the Binkit dataset. As shown in Figure 8, all models exhibit a decreasing trend in Recall@1 as the pool size increases. When the pool size is small, the Recall@1 values of all models are nearly 1.0. However, as the pool size grows, the Recall@1 performance of the GBsim model decreases more slowly. This indicates that as the pool size increases and the model performance declines, GBsim still maintains the highest Recall@1 performance. It implies that the two-stage search strategy plays an effective role in the function search scenario, achieving higher performance than methods that merely calculate the cosine distance of vectors (such as jTrans and SAFE). Although Asteria-Pro also employs a similar two-stage search strategy, the model architecture combining GCN and BERT exerts a more effective function compared to the method of extracting features from the abstract syntax tree.

The increasing pool size experiment serves as a progressive stress test for model robustness, as larger pools introduce more potential distractors and increase the difficulty of maintaining accurate matches. GBsim’s superior performance as pool size grows demonstrates its enhanced resilience to noise and distribution shifts in increasingly challenging search environments.

A detailed analysis in Table 1 reveals that GBsim outperforms its competitors in both the MRR and Recall@1 metrics with a pool size of 10,000. Specifically, GBsim achieves an average MRR of 0.901 and an average Recall@1 of 0.831. Compared to the best-performing baseline model, Asteria-Pro (MRR = 0.875, Recall@1 = 0.810), GBsim improves Recall@1 by 2.59 percentage points and MRR by 2.97 percentage points.

In particular, as the optimization span increases (from O0 to O3), the performance of all models exhibits a decreasing trend. This indicates that different optimization levels indeed introduce significant challenges to binary function comparison tasks. Despite these challenges, GBsim consistently maintains superior performance across all optimization levels, demonstrating its robustness and effectiveness in handling cross-optimization scenarios. This highlights the advantage of GBsim’s design, which leverages a cross-architecture language model and a hybrid layer combining GCN and BERT to extract both structural and semantic information of functions, resulting in high-quality binary function embeddings. This design enables GBsim to effectively capture high-quality function semantics in cross-architecture and cross-optimization scenarios, significantly enhancing the robustness and accuracy of binary code similarity analysis. From an information theory perspective, this suggests that GBsim’s hybrid architecture more effectively preserves the essential semantic and structural information that remains invariant across optimization transformations.

### 6.3. Real-World Vulnerability Scenarios(RQ3)

To rigorously evaluate GBsim’s performance in vulnerability detection, we constructed a dataset focused on vulnerabilities in OpenSSL version 1.0.1. Specifically, we meticulously selected 20 CVEs (Common Vulnerabilities and Exposures) from this version and pinpointed the corresponding vulnerable functions. During the data preprocessing phase, we ensured data quality by filtering out functions with compilation errors or an insufficient number of basic blocks. Subsequently, through cross-architecture and cross-optimization compilation techniques, we generated a function set comprising 142 binary functions, all sourced from vulnerable samples. Additionally, we constructed a complementary set of 358 functions from the same OpenSSL version that are not associated with any vulnerabilities, resulting in a pool of 500 functions in total.

For evaluation, 100 vulnerable functions were randomly sampled and compared against all functions in the pool. The highest similarity match for each comparison was used to calculate the recall rate. As summarized in Table 2, GBsim achieved a recall of 81.3%, surpassing the best baseline, jTrans (recall = 79.2%), by a margin of 2.1 percentage points. These results highlight GBsim’s remarkable advantage in vulnerability detection, demonstrating its superior recall performance compared to other baseline models. This strongly validates GBsim’s capability to precisely identify vulnerable functions in tasks involving OpenSSL version 1.0.1, further establishing its robustness in vulnerability detection. This suggests that GBsim could achieve even better performance in real-world vulnerability search tasks by replacing GCN and BERT with state-of-the-art GNN and NLP models.

### 6.4. Comparison of Efficiency: Balancing Robustness and Computational Cost (RQ4)

We evaluated the inference cost of GBsim in datasets of varying sizes and compared it with baseline models. Since different baseline models use distinct preprocessing methods and pretraining approaches (e.g., BERT-like models versus Word2Vec-based models), we excluded the time cost associated with model preparation. This exclusion is reasonable because these processes are typically performed only once during training and do not affect the inference phase. Our analysis focused solely on the inference cost of each model on the test set.

We measured the runtime on test datasets of different sizes. As shown in Table 3, GBsim’s inference time is comparable to that of jTrans and is slightly faster. GBsim employs a complex function embedding network and a comparison network, which results in slightly longer inference times compared to simpler models. However, this complexity is a necessary trade-off for its superior performance in vulnerability detection tasks, as demonstrated in our earlier experiments. If more advanced and efficient GNN and NLP components are integrated into GBsim, its inference efficiency could be further improved without compromising its accuracy.

This analysis reveals an important trade-off between computational efficiency and robustness in binary code similarity models. While GBsim requires slightly more computational resources than simpler models, this investment translates directly into significantly improved robustness across distribution shifts, making it a worthwhile compromise for critical security applications where reliability under varying conditions is paramount. From an information theory perspective, the additional computational cost can be viewed as the necessary resource investment to extract and process the invariant information that enables robust cross-architecture similarity detection.

### 6.5. The Effectiveness of the Two-Stage Search Approach (RQ5)

To validate the impact of the two-stage search approach on model performance, we designed an ablation experiment to verify its effectiveness. Using the GBsim model with the optimal hyperparameter combination, we established two control groups. The first group directly employs cosine similarity calculations on the vector representations of binary functions for the search task, named GBsim-cos. The second group uses a fully connected network (FCN) in its entirety with the same configuration as GBsim for comparison-based searching, named GBsim-FCN. Since the control groups do not involve the two-stage comparison, they are trained using the same training dataset as GBsim, without the need to train the two-stage comparison network. The experiments were carried out on a search test set with a pool size of 10,000. The results are shown in Table 4.

Table 4 presents a comparison of inference time (in seconds) and MRR for GBsim and its variants (GBsim-cos and GBsim-FCN) in a set of search tests with a pool size of 10,000. Specifically, GBsim achieves an inference time of 5.46 s and an MRR of 0.901; GBsim-cos has an inference time of 4.33 s and an MRR of 0.822, while GBsim-FCN exhibits an inference time of 7.94 s and an MRR of 0.905.

As shown in the table, GBsim shows superior performance in MRR, significantly outperforming GBsim-cos (MRR = 0.822), which relies solely on cosine similarity. Compared to GBsim-FCN (MRR = 0.905), which depends entirely on a fully connected network, GBsim achieves comparable performance with a shorter inference time. Although GBsim-FCN has a slightly higher MRR than GBsim, its inference time is significantly longer (7.94 s). In contrast, GBsim strikes a better balance between performance and efficiency through its two-stage search strategy. Specifically, GBsim sacrifices only approximately 0.44% in MRR (from 0.905 to 0.901) while achieving a 31.2% improvement in efficiency (reducing the inference time from 7.94 s to 5.46 s). This further validates the effectiveness of the GBsim design, which combines the rapid filtering of cosine similarity with the fine-grained comparison of a fully connected network, enabling high accuracy and significantly enhancing search efficiency. This design makes GBsim highly competitive in practical applications, particularly in large-scale binary function search tasks, where it can efficiently and accurately complete the task.

## 7. Discussion

The experimental results of GBsim not only validate its effectiveness in cross-architecture binary code similarity analysis but also provide important conclusions through systematic experiments, offering valuable insights for future research.

### 7.1. Experimental Conclusions

1. **Effectiveness of early model components.** The experiments of GBsim demonstrate that even early pioneering model components (such as GCN and BERT) can achieve excellent performance in binary code similarity analysis tasks, as long as they effectively combine structural information (extracted by GCN) and semantic information (learned by BERT). This conclusion suggests that the key to model design lies not in using the latest components but in how to effectively integrate the strengths of different feature extraction methods.
2. **Special characteristics of assembly language models.** In the construction of assembly language models, the experimental results of GBsim show that Word2Vec outperforms BERT. This may be because the optimizations of BERT in natural language processing (such as dynamic context and polysemy handling) are not fully applicable to assembly language. The precision and limited vocabulary of the assembly language make simpler embedding methods such as Word2Vec more advantageous. This finding provides important guidance for future choices in the design of assembly language models.
3. **Relationship between model complexity and performance.** The experiments also reveal that model performance does not improve monotonically with increasing complexity. The excessive stacking of layers (e.g., more than two GCN layers) may lead to performance degradation, especially in highly complex networks. This can be attributed to the over-smoothing effect in GNNs: most CFGs in binary code are small-scale, where excessive GCN layers cause indistinguishable node representations. This phenomenon highlights the need to strike a balance between model complexity and performance in binary code analysis model design, avoiding over-engineering.

### 7.2. Interpreting Results Through the Lens of Robustness

The experimental results of GBsim provide valuable insights into GNN robustness in binary code analysis. From an information theory perspective, the balance between model complexity and performance can be understood as finding the optimal point where the model captures sufficient information to make accurate predictions without overfitting to noise or architecture-specific details. The superior performance of simpler Word2Vec embeddings over BERT suggests that in domains with limited vocabulary and precise semantics, simpler models may extract more robust representations by focusing on the most essential information while discarding irrelevant variations. Additionally, the observed performance plateau with increasing GCN layers indicates that there exists an information saturation point beyond which additional structural processing introduces more noise than signal, potentially degrading the model’s robustness to distribution shifts.

### 7.3. Limitations of GBsim

Despite its outstanding performance in experiments, GBsim still has some limitations that need to be addressed in future research.

1. **High computational cost.** The hybrid architecture of GBsim (GCN+BERT) incurs computational overhead due to the inherent complexity of jointly processing graph structures and semantic sequences, which imposes GPU memory and latency constraints in large-scale deployments. While this limitation may affect real-time applications, our architectural choices were motivated by a methodological focus: to validate that structural and semantic fusion itself, even instantiated through foundational models, can fundamentally advance binary code embedding. Specifically, GCN and BERT serve as paradigmatic instantiations of structural graph modeling and contextual semantic representation, respectively. Their selection prioritizes conceptual transparency over computational optimization, establishing a lower-bound performance baseline for the proposed fusion paradigm. As both domains evolve (e.g., RoBERTa [28]’s optimized pretraining), adopting more efficient variants could mitigate these costs without altering the core methodology. Our contribution thus lies not in pushing model-specific efficiencies, but in demonstrating that structural-semantic synergy enables qualitatively new embedding capabilities.
2. **Limited generalization ability.** The performance of GBsim can be degraded in unfamiliar datasets, indicating there is room for improvement in its **robustness against extreme distribution shifts**. One potential solution is to construct more extensive datasets covering diverse compiler configurations, optimization levels, and instruction set architectures. Additionally, integrating domain adaptation techniques (e.g., feature alignment between seen/unseen ISAs) or contrastive learning objectives could enhance cross-architecture generalization. Leveraging large language models (e.g., GPT) to align binary/source code semantics may further boost out-of-distribution robustness through richer program understanding. While the current framework focuses on demonstrating cross-architecture feasibility, future extensions incorporating meta-learning paradigms could specifically address rapid adaptation to novel ISAs.

### 7.4. Future Directions

The success of GBsim provides new information for the analysis of binary code similarity, while its limitations point to future research directions. By optimizing model architectures (including explainable graph neural networks [30] for transparent similarity judgments), expanding datasets, and integrating large language models, we can enhance accuracy, efficiency, and operational trustworthiness, which are particularly critical in vulnerability detection scenarios where false positives may incur severe security consequences. These improvements will bring greater value to software security analysis through interpretable pattern discovery and risk-controlled decision making.

## 8. Conclusions

In this paper, we introduce GBsim, a novel approach to the embedding of binary functions that addresses key robustness challenges in the detection of binary code similarity. By integrating natural language processing techniques with graph neural networks, GBsim constructs a robust cross-architecture assembly language model and a multilayer hybrid network that remains stable despite significant distribution shifts between different architectures and optimization levels. Our extensive experiments on the BINKIT dataset demonstrate that GBsim significantly outperforms state-of-the-art baseline models across multiple metrics, including MRR and Recall@1, while also excelling in real-world vulnerability detection tasks.

Specifically, GBsim achieved an MRR of 0.901 and a Recall@1 of 0.831 with a pool size of 10,000, surpassing the best-performing baseline model, Asteria-Pro (MRR = 0.875, Recall@1 = 0.810), by 2.97 and 2.59 percentage points, respectively. This performance advantage becomes even more pronounced at larger pool sizes, highlighting GBsim’s scalability and robustness. In real-world vulnerability detection scenarios, GBsim achieved a recall rate of 81.3% on the OpenSSL 1.0.1 dataset, outperforming jTrans (recall = 79.2%) by 2.1 percentage points. These results underscore GBsim’s capability to precisely identify vulnerable functions, making it a powerful tool for real-world security applications.

The hyperparameter sensitivity analysis revealed that the optimal configuration of GBsim consists of a Word2Vec embedding model, a two-layer GCN, a one-layer BERT, and a three-layer hybrid architecture. This configuration strikes a balance between model complexity and performance, as evidenced by the diminishing returns observed with additional GCN layers in highly complex networks. Furthermore, our evaluation of inference costs showed that GBsim’s runtime is comparable to that of jTrans, with inference times of 7.12 s for a pool size of 10,000, despite its more complex architecture. This efficiency, combined with its superior accuracy, makes GBsim a practical solution for large-scale binary code analysis.

These findings demonstrate that GBsim not only achieves state-of-the-art performance in standard settings but also maintains exceptional robustness under challenging real-world conditions where distribution shifts between training and deployment environments are inevitable.

In summary, GBsim not only provides an effective solution for cross-architecture binary code similarity detection, but also offers valuable insights into the robustness of graph neural networks when applied to complex structural data with inherent noise and distribution shifts. By addressing these fundamental robustness challenges, GBsim establishes a new benchmark for reliable binary code analysis in security-critical applications.

## Figures and Tables

**Table 1:** Performance comparison of different models across optimization levels. MRR and Recall@1 of GBsim and baselines across different optimization levels (O0, O1, O2, O3) with Poolsize = 10,000. “Avg.” represents the average performance across all optimization level pairs.

	MRR				Recall@1			
	O0, O3	O1, O3	O2, O3	Avg.	O0, O3	O1, O3	O2, O3	Avg.
**SAFE**	0.381	0.698	0.724	0.691	0.352	0.644	0.707	0.603
**jTrans**	0.639	0.844	0.872	0.827	0.617	0.812	0.869	0.787
**Asteria-Pro**	0.726	0.897	0.924	0.875	0.709	0.851	0.872	0.810
**GBsim**	0.876	0.912	0.951	0.901	0.819	0.903	0.916	0.831

**Table 2:** Recall comparison of different models. Recall values for different models in real-world vulnerability.

	SAFE	jTrans	Asteria-Pro	GBsim
**recall**	0.238	0.792	0.768	0.813

**Table 3:** Inference time (in seconds) for different models across varying pool sizes.

Pool Size	SAFE	jTrans	Asteria-Pro	GBsim
100	0.04	0.12	0.08	0.11
1000	0.31	1.44	0.77	1.62
5000	1.22	4.29	3.27	4.03
10,000	2.03	8.93	5.46	7.12

**Table 4:** Performance and efficiency comparison of GBsim variants. Comparison of inference time (in seconds) and MRR for GBsim and its variants (GBsim-cos and GBsim-FCN) on a search pool size of 10,000.

	GBsim	GBsim-cos	GBsim-FCN
**time**	5.46	4.33	7.94
**MRR**	0.901	0.822	0.905

## Data Availability

The dataset used in this study was obtained from BINKIT [29]. The code for dataset processing, model training, and evaluation is publicly available on GitHub at https://github.com/kidding1412/GBsim (accessed on 2 February 2025), enabling other researchers to access and reuse it for further studies.

## References

[1] David Y., Partush N., Yahav E., Shen X., Tuck J., Bianchini R., Sarkar V. (2018). FirmUp: Precise Static Detection of Common Vulnerabilities in Firmware. Proceedings of the Twenty-Third International Conference on Architectural Support for Programming Languages and Operating Systems, ASPLOS 2018.

[2] Hu X., Shin K.G., Bhatkar S., Griffin K., Birrell A., Sirer E.G. (2013). MutantX-s: Scalable Malware Clustering Based on Static Features. Proceedings of the 2013 USENIX Annual Technical Conference, USENIX ATC 2013.

[3] Luo L., Ming J., Wu D., Liu P., Zhu S. (2017). Semantics-Based Obfuscation-Resilient Binary Code Similarity Comparison with Applications to Software and Algorithm Plagiarism Detection. IEEE Trans. Softw. Eng..

[4] Gao D., Reiter M.K., Song D.X., Chen L., Ryan M.D., Wang G. (2008). BinHunt: Automatically Finding Semantic Differences in Binary Programs. Proceedings of the Information and Communications Security, 10th International Conference, ICICS 2008.

[5] Ding S.H.H., Fung B.C.M., Charland P. Asm2Vec: Boosting Static Representation Robustness for Binary Clone Search against Code Obfuscation and Compiler Optimization. Proceedings of the 2019 IEEE Symposium on Security and Privacy (SP).

[6] Tian D., Jia X., Ma R., Liu S., Liu W., Hu C. (2021). BinDeep: A Deep Learning Approach to Binary Code Similarity Detection. Expert Syst. Appl..

[7] Duan Y., Li X., Wang J., Yin H. DeepBinDiff: Learning Program-Wide Code Representations for Binary Diffing. Proceedings of the Network and Distributed System Security Symposium.

[8] Xu X., Liu C., Feng Q., Yin H., Song L., Song D. Neural Network-based Graph Embedding for Cross-Platform Binary Code Similarity Detection. Proceedings of the 2017 ACM SIGSAC Conference on Computer and Communications Security.

[9] Zuo F., Li X., Young P., Luo L., Zeng Q., Zhang Z. (2019). Neural Machine Translation Inspired Binary Code Similarity Comparison beyond Function Pairs. Proceedings of the 26th Annual Network and Distributed System Security Symposium, NDSS 2019.

[10] Yu Z., Cao R., Tang Q., Nie S., Huang J., Wu S. (2020). Order Matters: Semantic-Aware Neural Networks for Binary Code Similarity Detection. Proceedings of the Thirty-Fourth AAAI Conference on Artificial Intelligence, AAAI 2020.

[11] Wang H., Gao Z., Zhang C., Sun M., Zhou Y., Qiu H., Xiao X. CEBin: A Cost-Effective Framework for Large-Scale Binary Code Similarity Detection. Proceedings of the ACM SIGSOFT International Symposium on Software Testing and Analysis (ISSTA 2024).

[12] Liu B., Huo W., Zhang C., Li W., Li F., Piao A., Zou W., Huchard M., Kästner C., Fraser G. (2018). *α*Diff: Cross-Version Binary Code Similarity Detection with DNN. Proceedings of the 33rd ACM/IEEE International Conference on Automated Software Engineering, ASE 2018.

[13] Lageman N., Kilmer E.D., Walls R.J., McDaniel P.D., Deng R.H., Weng J., Ren K., Yegneswaran V. (2016). BinDNN: Resilient Function Matching Using Deep Learning. Proceedings of the Security and Privacy in Communication Networks—12th International Conference, SecureComm 2016.

[14] Li Y., Gu C., Dullien T., Vinyals O., Kohli P., Chaudhuri K., Salakhutdinov R. (2019). Graph Matching Networks for Learning the Similarity of Graph Structured Objects. Proceedings of the 36th International Conference on Machine Learning, ICML 2019.

[15] Massarelli L., Luna G.A.D., Petroni F., Baldoni R., Querzoni L., Perdisci R., Maurice C., Giacinto G., Almgren M. (2019). SAFE: Self-Attentive Function Embeddings for Binary Similarity. Proceedings of the Detection of Intrusions and Malware, and Vulnerability Assessment—16th International Conference, DIMVA 2019.

[16] Massarelli L., Luna G., Petroni F., Querzoni L. Investigating Graph Embedding Neural Networks with Unsupervised Features Extraction for Binary Analysis. Proceedings of the Workshop on Binary Analysis Research (BAR) 2019.

[17] Wang H., Qu W., Katz G., Zhu W., Gao Z., Qiu H., Zhuge J., Zhang C., Ryu S., Smaragdakis Y. (2022). jTrans: Jump-Aware Transformer for Binary Code Similarity Detection. Proceedings of the ISSTA ’22: 31st ACM SIGSOFT International Symposium on Software Testing and Analysis.

[18] Pei K., Xuan Z., Yang J., Jana S., Ray B. (2020). Trex: Learning Execution Semantics from Micro-Traces for Binary Similarity. arXiv.

[19] Kipf T.N., Welling M. (2016). Semi-Supervised Classification with Graph Convolutional Networks. arXiv.

[20] Devlin J., Chang M.W., Lee K., Toutanova K. BERT: Pre-training of Deep Bidirectional Transformers for Language Understanding. Proceedings of the 2019 Conference of the North American Chapter of the Association for Computational Linguistics: Human Language Technologies, NAACL-HLT 2019.

[21] Mikolov T., Chen K., Corrado G., Dean J. Efficient Estimation of Word Representations in Vector Space. Proceedings of the 1st International Conference on Learning Representations, ICLR 2013.

[22] Yang S., Dong C., Xiao Y., Cheng Y., Shi Z., Li Z., Sun L. (2024). Asteria-Pro: Enhancing Deep Learning-based Binary Code Similarity Detection by Incorporating Domain Knowledge. ACM Trans. Softw. Eng. Methodol..

[23] Bai Y., Ding H., Bian S., Chen T., Sun Y., Wang W., Culpepper J.S., Moffat A., Bennett P.N., Lerman K. (2019). SimGNN: A Neural Network Approach to Fast Graph Similarity Computation. Proceedings of the Twelfth ACM International Conference on Web Search and Data Mining, WSDM 2019.

[24] Gao J., Yang X., Fu Y., Jiang Y., Sun J. VulSeeker: A Semantic Learning Based Vulnerability Seeker for Cross-Platform Binary. Proceedings of the 2018 33rd IEEE/ACM International Conference on Automated Software Engineering (ASE).

[25] Wang H., Gao Z., Zhang C., Sha Z., Sun M., Zhou Y., Zhu W., Sun W., Qiu H., Xiao X. CLAP: Learning Transferable Binary Code Representations with Natural Language Supervision. Proceedings of the ACM SIGSOFT International Symposium on Software Testing and Analysis (ISSTA 2024).

[26] Wang L., He D., Zhang H., Liu Y., Wang W., Pan S., Jin D., Chua T., Wooldridge M.J., Dy J.G., Natarajan S. (2024). GOODAT: Towards Test-Time Graph Out-of-Distribution Detection. Proceedings of the Thirty-Eighth AAAI Conference on Artificial Intelligence, AAAI 2024.

[27] Luo Z., Wang P., Wang B., Tang Y., Xie W., Zhou X., Liu D., Lu K. (2023). VulHawk: Cross-architecture Vulnerability Detection with Entropy-based Binary Code Search. Proceedings of the 30th Annual Network and Distributed System Security Symposium, NDSS 2023.

[28] Liu Y., Ott M., Goyal N., Du J., Joshi M., Chen D., Levy O., Lewis M., Zettlemoyer L., Stoyanov V. (2019). RoBERTa: A Robustly Optimized BERT Pretraining Approach. arXiv.

[29] Kim D., Kim E., Cha S.K., Son S., Kim Y. (2022). Revisiting Binary Code Similarity Analysis Using Interpretable Feature Engineering and Lessons Learned. IEEE Trans. Softw. Eng..

[30] Zhang H., Wu B., Yuan X., Pan S., Tong H., Pei J. (2024). Trustworthy Graph Neural Networks: Aspects, Methods, and Trends. Proc. IEEE.

